# Comparison of microsatellites, single-nucleotide polymorphisms (SNPs) and composite markers derived from SNPs in linkage analysis

**DOI:** 10.1186/1471-2156-6-S1-S29

**Published:** 2005-12-30

**Authors:** Chao Xing, Fredrick R Schumacher, Guan Xing, Qing Lu, Tao Wang, Robert C Elston

**Affiliations:** 1 Department of Epidemiology and Biostatistics, Wolstein Research Building, Case Western Reserve University, Cleveland, OH 44106, USA

## Abstract

There is growing evidence that a map of dense single-nucleotide polymorphisms (SNPs) can outperform a map of sparse microsatellites for linkage analysis. There is also argument as to whether a clustered SNP map can outperform an evenly spaced SNP map. Using Genetic Analysis Workshop 14 simulated data, we compared for linkage analysis microsatellites, SNPs, and composite markers derived from SNPs. We encoded the composite markers in a two-step approach, in which the maximum identity length contrast method was employed to allow for recombination between loci. A SNP map 2.3 times as dense as a microsatellite map (~2.9 cM compared to ~6.7 cM apart) provided slightly less information content (~0.83 compared to ~0.89). Most inheritance information could be extracted when the SNPs were spaced < 1 cM apart. Comparing the linkage results on using SNPs or composite markers derived from them based on both 3 cM and 0.3 cM resolution maps, we showed that the inter-SNP distance should be kept small (< 1 cM), and that for multipoint linkage analysis the original markers and the derived composite markers had similar power; but for single point linkage analysis the resulting composite markers lead to more power. Considering all factors, such as information content, flexibility of analysis method, map errors, and genotyping errors, a map of clustered SNPs can be an efficient design for a genome-wide linkage scan.

## Background

Traditionally, genome-wide linkage scans employ low-density maps of microsatellite markers, or short tandem repeat polymorphisms (STRPs), spaced at intervals of ~10 cM across the genome. Although single-nucleotide polymorphisms (SNPs) are less informative than STRPs, they are distributed densely and uniformly throughout the genome, which can make up for their lack of informativeness. Moreover, SNP genotyping is easily automated, cost-effective, and low in error rate [1]. Genome-wide linkage scans tend to employ high density maps of SNPs because both theoretical and simulation studies [2-5], as well as real data applications [e.g., [6]], indicate that SNPs can achieve superior power to detect and localize linkage.

Because the power of a linkage study increases with the markers' information content (IC), comparison between SNP and STRP maps for linkage has mostly been focused on IC. When SNPs are uniformly distributed along the genome, multipoint analysis of dense SNPs can provide linkage IC comparable to that of less dense STRPs. To obtain equivalent IC, the ratio of the number of SNPs to STRPs has been estimated to be 1.7–2.5 [2,4]. When the map is made up of clusters of SNPs spaced at intervals similar to those in a STRP map, several tightly linked SNPs considered as a single composite marker can provide linkage IC comparable to that of a highly informative STRP. Wilson and Sorant [3] showed this equivalence by comparing the power to detect linkage using each type of marker, and Goddard and Wijsman [4] did so by proposing a new measure of multilocus polymorphic information content (MPIC).

The Genetic Analysis Workshop 14 (GAW14) simulated data mimic a genome scan of a behavioral disorder with a genome scan map of STRPs ~7.5 cM apart, a genome scan map of SNPs ~3 cM apart, and a fine map of SNPs ~0.3 cM apart. Thus, we have an opportunity to compare STRPs and SNPs in genome-wide linkage analysis. There are two specific aims in this paper: 1) to compare the IC provided by STRPs, evenly spaced SNPs, and composite markers derived from tightly linked SNPs; and 2) to investigate the influence of inter-SNP distance on linkage analysis.

## Methods

Replicate 33 of the 100 Karangar nuclear pedigrees was randomly chosen from the GAW14 simulated data. We analyzed chromosomes 1, 3, 5, and 9, at which the simulated disease susceptibility loci lie. In addition to the STRP map and the 3-cM SNP map, we also "purchased" 2 packages of 0.3-cM SNPs that spanned the regions covering the disease susceptibility loci on each chromosome. Specifically, packages 028, 029 (38 SNPs), packages 153, 154 (26 SNPs), packages 207, 208 (38 SNPs), and packages 417, 418 (38 SNPs) were purchased for chromosome 1, 3, 5, and 9, respectively.

For a cluster of tightly linked SNPs, haplotypes are analogous to the alleles of a STRP marker, and thus the whole cluster forms a composite marker. A recombination within a cluster can lead to Mendelian inconsistency of genotypes. To avoid this type of inconsistency, and to study the influence of inter-SNP distance on linkage analysis, we encoded the composite markers in a two-step approach. First, we generated the most likely haplotype for every family member based on the SNP data and the given recombination fraction between consecutive pairs of SNPs using the software MERLIN [7] and encoded the founders' composite marker genotypes according to their haplotypes. Second, the non-founders' composite marker genotypes were determined by comparing the similarity between the founders' and non-founders' haplotypes using the maximum identity length contrast (MILC) method [8]. Let *S*(*i*) denote the score of identity length at locus *i*. If the two alleles at the *i*^th ^SNP are different, *S*(*i*) = 0; if they are identical in state (IIS), we repeat the comparison process for the next SNP on each side, and this is repeated to determine *S*(*i*). After the *S*(*i*) values were calculated at each SNP between any pair of founder and non-founder haplotypes, every 3 (or 5) SNPs were grouped into a cluster as one composite marker and a mean score  was calculated for each cluster. The largest mean score was then used to assign haplotypes. Suppose, for example, that for a particular trio at a given cluster, *P*_1 _and *P*_2 _denote the father's two haplotypes, *M*_1 _and *M*_2 _the mother's two haplotypes, and *O*_1 _and *O*_2 _the child's two haplotypes. If the largest mean score was for the *P*_1 _- *O*_1 _pair, then the child inherited the haplotype *P*_1 _and the corresponding composite marker allele; the other haplotype inherited was then *M*_1 _or *M*_2 _depending on which pair (*M*_1 _- *O*_2 _versus *M*_1 _- *O*_2_) had the larger score. If the largest scores for *P*_1 _- *O*_1 _and *M*_1 _- *O*_1 _were equal, then *O*_1 _was randomly assigned to be from either parent. The map position of a composite marker was labelled as being in the middle of the cluster of SNPs.

The multipoint IC, measuring the fraction of inheritance information extracted by the map relative to that extracted by an infinitely dense polymorphic map [2], is based on the entropy of the probability distribution of inheritance vectors [9]. The IC was calculated by the program MLOD. Both single-point and multipoint linkage analysis of being affected with Kofendred Personality Disorder was performed by the Haseman-Elston method [10-12] as implemented by option w4 in the program SIBPAL. Single-point and multipoint IBD-sharing estimates for SNPs and composite markers were calculated by the program GENIBD. These programs are included in the S.A.G.E. software suite, version 5.0, 2004 [13].

## Results

Table 1 displays the IC corresponding to different inter-marker distances for STRPs, SNPs, and composite markers with 3 or 5 SNPs in a cluster. For nuclear families with all members' genotypes known, a SNP map 2.3 times as dense as a SRTP map (~2.9 cM compared to ~6.7 cM apart) provided slightly less IC than the SRTP map (~0.83 compared to ~0.89). The majority of the inheritance information (~0.98) could be extracted when the SNPs were spaced ~0.30 cM apart. There was a slight increase in IC (~0.86 compared to ~0.83) when 3 SNPs were grouped into a cluster (spaced ~9.6 cM apart) in the 3-cM SNP map; however, the opposite trend was observed when grouping 5 SNPs into a cluster (spaced ~16 cM apart), except for chromosome 9. There was also a slight increase in IC (~0.99 compared to ~0.98) when 3 or 5 SNPs were grouped into a cluster (spaced ~0.91 or ~1.5 cM apart) in the 0.3-cM SNP map.

**Figure 1:**
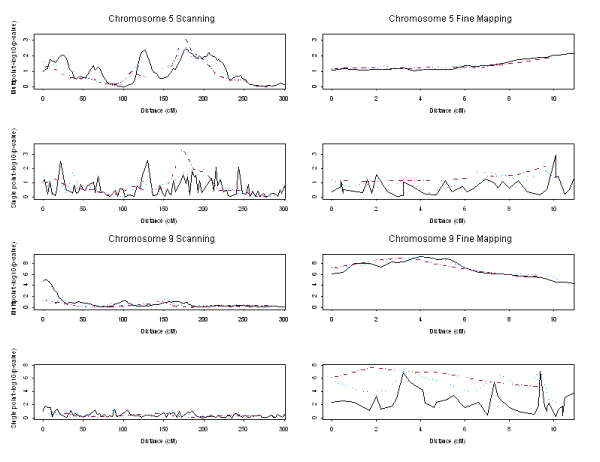
**Single-point and multipoint linkage signals by Haseman-Elston regression. **Scanning indicates using the map of SNPs ~3 cM apart; fine mapping indicates using the map of SNPs ~0.3 cM apart. Solid line: single SNP as a marker; dotted line: 3 SNPs in a cluster; dashed line: 5 SNPs in a cluster.

Figure 1 displays both the single point and multipoint linkage signals in terms of -log_10 _(*p*-value) by Haseman-Elston regression. Here we only report the results for chromosomes 5 and 9, because there was no signal reaching nominal significance (*p*-value ≤ 5 × 10^-2^) for chromosome 1 or 3 in this replicate. For chromosome 5, at the simulated disease susceptibility locus (~3.2 cM) only multipoint and single point analyses using 3-SNP markers from the 3-cM map detected linkage signals with *p*-values less than 5 × 10^-2 ^(2 × 10^-2 ^and 3 × 10^-2^, respectively). Both multipoint and single-point analyses using 1-, 3-, and 5-SNP markers from the 3-cM and 0.3 cM maps generated false linkage signals at other locations. For chromosome 9, at the simulated disease susceptibility locus (~3.5 cM) multipoint analyses using 1- and 3-SNP markers from the 3-cM map detected linkage signals with *p*-values of 2 × 10^-5 ^and 3 × 10^-2^, respectively; single-point analyses detected linkage signals at the same position with p-values of 1 × 10^-2 ^and 4 × 10^-2^, respectively. Analyses using 5-SNP markers did not detect linkage signals with *p*-values less than 5 × 10^-2^. When employing the 0.3-cM map, each analysis detect the designed linkage with *p*-value less than 1 × 10^-5^. When using the 3-cM map, the single point analysis had weak power to detect linkage because of the low informativeness of a single SNP; composite markers could not make any improvement – they even resulted in loss of signal on chromosome 9 by multipoint analysis. When using the 0.3-cM map, both composite markers and single SNPs gained power, and gave quite similar results with multipoint analysis. When employing the single-point approach, the composite markers produced higher and smoother signals than did the single SNPs.

## Discussion

The relationship between the IC of SNP and STRP maps is not simple [14]. To achieve the same amount of information, Kruglyak [2] speculated that the ratio of the equivalent number of SNPs to STRPs is 2.25 to 2.5 in first-cousin pairs, and Goddard and Wijsman [4] speculated that the ratio is 1.7 in nuclear families. On the basis of the GAW14 simulated data, we found that the SNP map provided slightly less IC when the ratio was 2.3, different from former studies. Based on real data, Matise et al. [14] found the ratio to be 2.76 on chromosome 12; however, they also noticed that the ratio changed with many factors. Family structure and knowledge of parental genotypes may play important roles in this.

IC varies as a function of SNP density. The denser the map, the more IC can be extracted. In this study of nuclear families with parental genotypes known, the 3-cM map gave an IC of 0.83 and the 0.3-cM map gave an IC of 0.98. Together with the observations of Evans and Cardon [5] that increasing the density of SNPs within a 1-cM map had little effect on IC when parental genotypes are known, we conclude that, if parents can be genotyped, a SNP map of resolution ~1 cM/SNP should suffice to infer inheritance patterns.

The recombination between loci in a cluster is usually ignored, given tight linkage. Wilson and Sorant [3] simulated distances between SNPs of 2 cM, and discarded the pedigree if any recombination occurred within a cluster, which diminished the power of composite markers. The MILC method is tolerant to recombination when there is tight linkage, and thus gains full power for composite markers. In the case of the 0.3-cM map, the composite markers behaved similarly to evenly spaced SNPs with multipoint analysis, and better than evenly spaced SNPs with single-point analysis. In the case of the 3-cM map, however, the composite markers were not better with single-point analysis, and even lost the signal on chromosome 9 with multipoint analysis. One possible reason for signal loss is that the susceptibility locus was at the left end of chromosome 9, where the MILC could not borrow much information from neighboring SNPs. In any case, when the inter-SNP distance is small (< 1 cM), one can employ the MILC method to take care of recombination, and then single-point linkage analysis has more power. This method can be applied to real data to construct composite markers. There are two aspects in which simulated data can be different from real data. First, there were no missing genotypes in the simulated data, while real data might have missing data. However, founders' missing genotypes will be imputed when we reconstruct the haplotypes, and a single marker can be skipped if there is any member missing that genotype. Second, the simulated data were all nuclear families, while real data might have multiple generations. However, after haplotype reconstruction we can recode the composite markers generation by generation using the same method we used for two generation pedigrees.

A clustered map structure can be more useful than a uniform SNP map for linkage analysis from practical consideration [4]. The clustered map structure can be relatively robust to map errors. Misspecifying inter-marker distance in multipoint linkage analyses can result in both power loss [15] and inflated type I error [16]. The accuracy of a dense map in terms of order and distance is problematic; however, the accuracy of a clustered map will be similar to that of a SRTP map with the effects of single map errors diluted. It is difficult to detect SNP genotyping errors by checking Mendelian inheritance; however, the effects of single genotyping errors can be minor in the context of a cluster of SNPs. Taking also into consideration the computation burden and superiority of single point linkage method for model-based analyses, a map of clustered SNPs can be an efficient design for a linkage genome scan.

## Abbreviations

GAW: Genetic Analysis Workshop

IBD: Identical by descent

IC: Information content

IIS: Identical in state

LD: Linkage disequilibrium

MILC: Maximum identity length contrast

MPIC: Multilocus polymorphic information content

SNP: Single-nucleotide polymorphism

STRPs: Short tandem repeat polymorphisms

## Authors' contributions

CX, FRS, and RCE conceived the study, and participated in its design and coordination. CX, FRS, and GX carried out programming and analyzed chromosomes 1 and 3. QL analyzed chromosome 5, and TW analyzed chromosome 9. All authors read and approved the final manuscript.

## Figures and Tables

**Table 1:** Mean Inter-marker distance and IC for STRPs, SNPs, and composite Markers

	Mean intermarker distance in centimorgans (mean IC)			
	
Map	Chr 1	Chr 3	Chr 5	Chr 9
STRP	6.90 (0.88)	6.80 (0.88)	6.54 (0.89)	6.74 (0.89)
3-cM SNP				
1-SNP^a^	2.96 (0.81)	2.98 (0.83)	2.80 (0.85)	2.90 (0.82)
3-SNP^b^	9.83 (0.83)	9.66 (0.85)	9.29 (0.88)	9.59 (0.88)
5-SNP^c^	16.39 (0.81)	16.11 (0.83)	15.54 (0.84)	16.00 (0.93)
0.3-cM SNP				
1-SNP^a^	0.34 (0.98)	0.25 (0.97)	0.31 (0.97)	0.29 (0.98)
3-SNP^b^	0.98 (0.99)	0.79 (0.98)	0.93 (0.98)	0.92 (0.99)
5-SNP^c^	1.58 (0.99)	1.26 (0.99)	1.64 (0.99)	1.55 (0.99)

^a ^Evenly spaced SNPs

^b ^3 SNPs in a cluster

^c ^5 SNPs in a cluster
